# Acute adaptations in dynamic torque and torque control to practicing with known and unknown sequences of concentric and eccentric muscle contractions in healthy young adults

**DOI:** 10.1186/s13102-026-01976-3

**Published:** 2026-08-14

**Authors:** Balázs Gáspár, Sarolta Tar, Ádám Fésüs, Patrik Ivusza, Mátyás Varga, Balázs Sebesi, Zsolt Murlasits, Anikó Kőnig-Péter, Tibor Hortobágyi, Márk Váczi

**Affiliations:** 1 Doctoral School of Biology and Sport Biology, Faculty of Sciences, University of Pécs, Pécs, Hungary; 2 Sports Medicine Center, Medical School, University of Pécs, Pécs, Hungary; 3 Institute of Sport Science and Physical Education, Faculty of Sciences, University of Pécs, Pécs, Hungary; 4 Institute of Bioanalysis, Medical School, University of Pécs, Pécs, Hungary; 5 Department of Kinesiology, Hungarian University of Sports Science, Budapest, Hungary; 6 Faculty of Sport Science, Ningbo University, Ningbo, China; 7 Department of Human Movement Sciences, University Medical Center, University of Groningen, Groningen, Netherlands

**Keywords:** Knee extensor, Dynamometer, Eccentric, Concentric, Mechanical work

## Abstract

**Background:**

During human movement, the neuromuscular system continuously adapts to varying and differently predictable demands. Practicing with sequences of known and unknown types of muscle contractions could train force control of muscle. We compared the acute effects of practicing with eccentric and concentric quadriceps muscle contractions at different velocities with known and unknown sequences, on knee extension torque, torque steadiness, and joint position sense in healthy young adults.

**Methods:**

Nineteen physically active young men practiced unknown and known muscle loading conditions on an isokinetic dynamometer, comprising eccentric and concentric contractions at different velocities in randomized and predefined sequences (16 contractions per condition). Joint position sense, maximal voluntary isometric contraction torque, and torque steadiness before and after practices, as well as mechanical work during the practice contractions were measured.

**Results:**

Maximal voluntary isometric contraction torque and joint position sense remained unchanged in both practice conditions, while torque steadiness variable error obtained at 70% of maximal voluntary isometric contraction torque decreased after known muscle loading. During eccentric and concentric practice contractions, mechanical work increased from trial to trial during known muscle loading. This improvement was limited to slow velocity concentric contraction after unknown muscle loading.

**Conclusion:**

The acute effects of using known and unknown muscle loadings were limited. Only known loadings improved torque steadiness, suggesting more efficient motor unit control. The trial-to-trial mechanical work adaptations observed mainly in known muscle loadings suggest rapid neuromuscular adjustments influenced by load predictability, rather than uncertainty.

## Introduction

During daily voluntary movements, humans experience unexpected disturbances that affect the smooth and successful execution of the movement. Unexpected perturbations can occur in many contexts, involving pushes, slips, or a loss of balance. For example, stepping down onto a lower‑than‑expected stair imposes an unexpected eccentric load on the lower‑limb muscles, whereas stepping up onto a higher‑than‑expected stair induces an unexpectedly strong concentric contraction of the leg muscles. Repeated exposures to such perturbations induce adaptations which can be quantified by measuring changes in proprioception, and motor performance [1–3], reflex latencies and amplitudes [4], adjustments in anticipatory feed-forward control [5, 6] and changes in stiffness control of the muscle-tendon unit [7]. Therapists and kinesiologists might be able to use this information to develop and guide novel therapies and prescribe exercise training.

In addition to a perturbation being unexpected, the type or nature of a motor disturbance can be also unknown to the subject [8–10]. In the stair climbing example, the perturbation is unexpected but the context still provides enough clues about details of the task. One way to create experimental perturbations in which the nature of the disturbance is not only unexpected but is also unknown is by varying the type and velocity of muscle contraction in an isokinetic setup [11]. By varying contraction type and contraction velocity, muscles can be exposed to a wide range of mechanical loads. Eccentric compared with concentric contractions generally produce greater muscle loadings [12, 13]. Variations in velocity also interact with contraction type by either augmenting or attenuating muscle tension, known as the principle of the Hill force-velocity relationship [14].

Eccentric and concentric contractions make up natural human movement cycles but often can occur unexpectedly so that we are unaware of the type of the oncoming contraction. Previous studies examining eccentric and concentric contractions showed that neural and mechanical responses are contraction-specific in EMG and torque. Differences were also found between eccentric and concentric training, as the eccentric group made greater errors in joint position perception and force control ability [15]. A sensitive measure of responses evoked by unknown eccentric and concentric muscle contractions is proprioception because it reflects changes in afferent feedback from muscle spindles and Golgi tendon organs, which are differentially modulated during muscle lengthening and shortening, thereby influencing sensorimotor control and force regulation [16]. Another outcome that could provide insights into understanding response adaptability to such perturbations is muscle force steadiness because it reflects the ability of the nervous system to regulate motor unit discharge variability and neural drive-in response to altered sensory input and motor demands [17, 18]. Force steadiness is also influenced by the type of muscle contraction performed, as eccentric contractions have been shown to impair submaximal force control to a greater extent, highlighting the specific challenges they pose to neuromuscular regulation [19]. Furthermore, the motor system can regulate the immediate responses to external perturbations by modulating stiffness [20]. In the presence of repeated perturbations, this immediate control mechanism allows the motor system to modify motor commands based on errors from previous trials, thereby reducing response errors. Moreover, a quick, trial-to-trial adaptation in movement quality (increased EMG activity, decreased time to perform the task) during unexpected loading is evident [21]. Therefore, the benefits of short-term practice with unknown compared with known type of muscle contraction (unknown muscle loading, UML; known muscle loading, KML) is that such training might induce immediate improvements in joint position sense (JPS), force steadiness, and muscle contractility. This hypothesis is further strengthened by substantial scientific evidence that both proprioceptive markers and muscle strength are critical for adaptation to mechanical perturbations, and that deficiency in these markers markedly impair adaptation potential [16, 22–26].

Therefore, the purpose of the present study was to compare the acute effects of practicing with eccentric and concentric quadriceps contractions at different velocities with known and unknown sequences, on knee extension torque, torque steadiness, and JPS in healthy young adults. We hypothesized that acute adaptations in force control would differ between sequences of repeated load perturbations that are known versus unknown to the subject. By contrasting these two practice conditions, we aim to gain insights into how healthy humans adjust to changing mechanical demands and if such practice environment might have a potential for a novel type of training and rehabilitation paradigm.

## Methods

### Participants

We recruited 19 physically active young men from the physical education and sport science student population at the local university (age: 24.2 ± 2.8 yrs; height: 181.2 ± 9.0 cm; sport participation: 13.5 ± 5.0 yrs; average training time: 4.9 ± 1.7 h/w). They had experience in resistance training but never used an isokinetic dynamometer before. Subject candidates were excluded who reported previous lower extremity injuries and knee surgeries. All subjects gave written informed consent according to the Declaration of Helsinki after receiving both a verbal and a written explanation of the experimental protocol and its potential risks. The University Ethics Committee approved the protocol (approval number: 7961). An a priori power analysis was performed using G*Power 3.1.9.7. for a repeated-measures ANOVA with within-subject factors. Assuming an effect size of f = 0.4, an alpha level of 0.05, a statistical power of 0.80, the required sample size was estimated at 15 participants. Finally, a total of 19 participants completed both practice conditions (UML and KML) with pre- and post-measurements.

### Experimental protocol

**Fig. 1:**
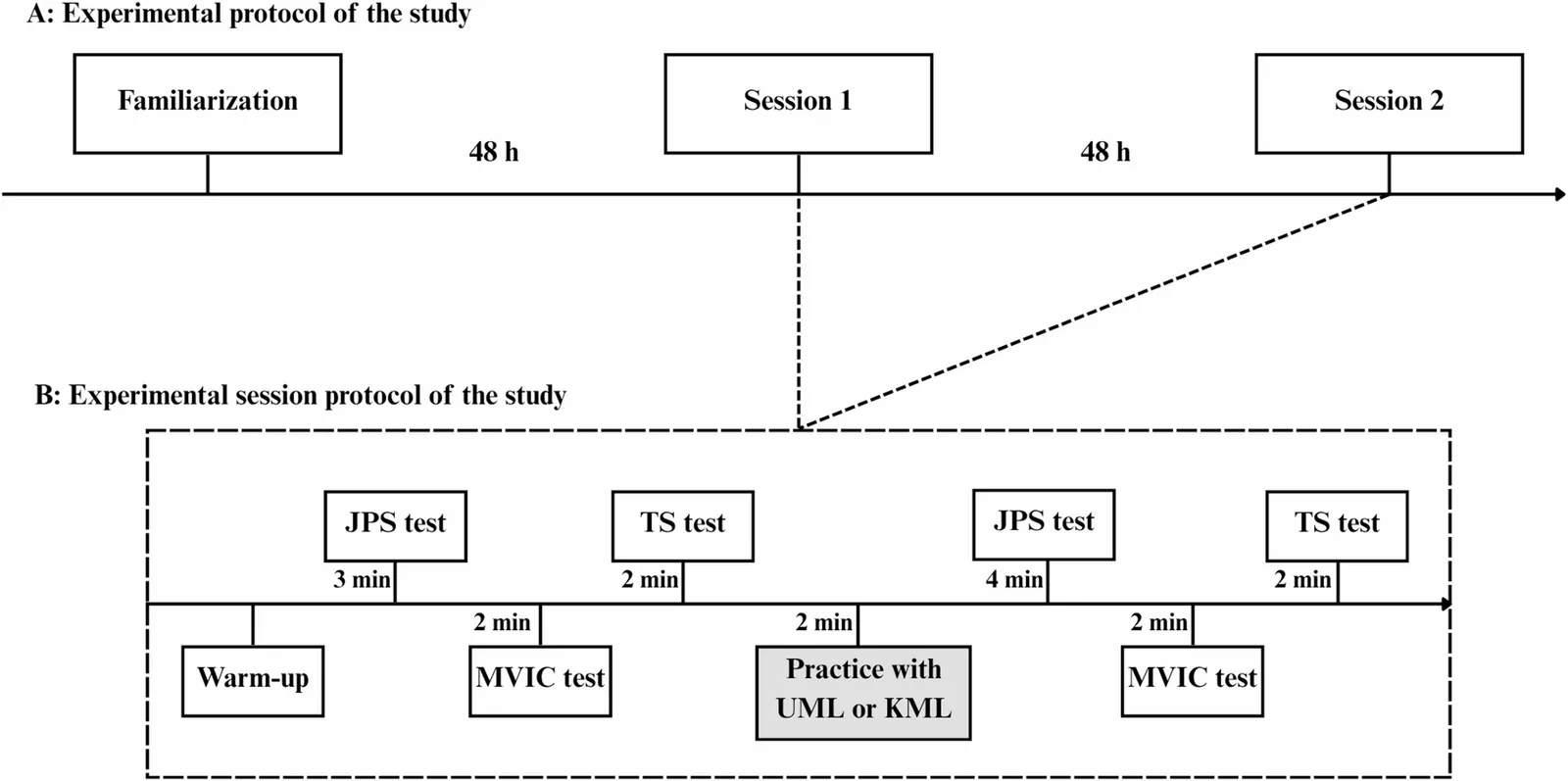
Overview of the experimental protocol (**A**) and experimental session protocol (**B**) of the study. Abbreviations: JPS = joint position sense, TS = torque steadiness, MVIC = maximal voluntary isometric contraction, UML = unknown muscle loading, KML = known muscle loading

Figure 1 shows the randomized crossover study design. Participants presented in the laboratory for one familiarization session, and two experimental sessions. The two experimental sessions served for conducting the test batteries and to practice the knee extensions with UMLs and KMLs. In the familiarization session, participants performed 3–4 submaximal isometric knee extensions, gradually increasing the contraction intensity from approximately 60 to 90% of their perceived maximal voluntary effort. They did not practice the UML or KML protocols. The experimental sessions began with a standardized 5-minute warm-up, including 3 min of cycling on a cycle ergometer (at a fixed power output of 60 Watts, between 50 and 70 rpm) and 2 min of dynamic stretching of the knee extensors consisting of walking quadriceps stretches, forward–backward leg swings, side-to-side leg swings, and walking lunges, each performed for 10 repetitions per leg. After warm-up, participants performed a passive knee JPS test, followed immediately by knee extension maximal voluntary isometric contraction (MVIC) test, and two minutes later, a knee extensor torque steadiness test with one leg, using isokinetic dynamometry. After two minutes of rest, with the same leg, participants performed either the UML or KML protocol using the same dynamometer. Four minutes after completing the practice protocol, the JPS, MVIC, and TS assessments were repeated (post-test) in the same order as during the pre-test assessment. Forty-eight hours later, during experimental session 2, the same procedure was repeated on the other leg, using the other muscle loading condition (either UML or KML). The order of the muscle loading conditions (UML and KML) and the order of the tested leg (dominant or non-dominant) were counterbalanced. Leg dominance was determined in the familiarization session by asking participants which leg they would kick a ball with.

### Joint position sense (JPS) test

The Multicont II dynamometer (Mediagnost, Budapest and Mechatronic Ltd., Szeged, Hungary) served for all tests and the muscle loading interventions in the present experiment. Detailed description of the technical specifications, operation, and calibration procedures have been previously published [27]. Participants were seated with the knee joint aligned with the axis of the dynamometer. The mid-thigh and the chest were secured with a pad and a velcro strap, respectively, and the distal leg was fastened to the lever arm, also with a velcro strap.

For JPS testing, we followed the methodology by Zhang et al. [28]. JPS was measured at 30°, 35°, 40°, 45°, 50°, 55°, 60° knee angles. The lever arm starting position was set at 80°, and the velocity at 5°/s for all measurements. The lever arm stopped at each preprogrammed joint angle for 5 s. Participants were not informed about the target joint angle but were instructed to memorize it during the 5-s period, with their eyes closed, so that no visual feedback was received about the position of the lever arm. Then, the lever arm returned to the 80° starting position at a velocity of 20°/s. The lever arm started to move again at 5°/s velocity and the participants stopped the movement with a press of a control knob at the memorized joint angle. The joint angle where the participants stopped the lever arm was recorded. The order of preprogrammed joint angles was randomized to eliminate learning effect during testing.

### Maximal Voluntary Isometric Contraction (MVIC)

Before the knee extension MVIC test, participants performed submaximal warm-up trials at ~ 70% and ~ 90% of perceived maximum. Measurements were performed at 50° knee joint angle. Participants performed the MVIC with their eyes open while receiving strong verbal encouragement. Two 5 s MVICs were executed with one minute rest between trials. Torque was recorded and the better value was considered for determining the target torque used in the torque steadiness test.

### Torque steadiness (TS) test

Similarly, to MVIC torque, TS was measured isometrically at the same knee joint angle. In the TS test, we considered the 20% and 70% target torques of the torque attained during the knee extension MVIC [29]. Participants were instructed to reach the predetermined knee extension torque within 3 s and maintain the target torque as steadily as possible, with minimal fluctuations, for the subsequent 10 s. Participants received visual feedback in the form of the real-time torque value displayed on a monitor. No target line was provided. Two 20% trials were performed first, which was followed by two 70% trials. There were two minutes rest among trials.

### Practice with UMLs and KMLs

In our practice protocol, we developed muscle loading stimulus variations by using isokinetic eccentric and concentric contractions, and by manipulating angular velocities. The concept is, as introduced earlier, that different muscle tension develops at eccentric vs. concentric contraction, and at low vs. fast velocity [30]. Therefore, the contraction variations and their characteristics were as follows for the UML condition when the subjects were unaware the contraction type: eccentric at 50°/s (EC50), eccentric at 250°/s (EC250), concentric at 50°/s (CC50), and concentric at 250°/s (CC250) angular velocity. The order of these contractions were randomized and this random order was fixed across subjects as follows: CC50, EC250, EC50, CC50, CC250, EC50, CC250, EC250 in the first set, and after EC250, CC50, CC250, EC250, EC50, CC250, EC 50, CC50 in the second set, giving a total of 16 repetitions in 2 sets. Between sets, 2 min rest was allowed. For every contraction, the dynamometer’s lever arm was driven to a starting position of 50° knee angle, while participants relaxed their knee extensors. Then, participants were instructed to initiate knee extension with maximum effort and velocity. As soon as the participant achieved the pre-set trigger threshold of 50 Nm, the lever rotated into the programmed direction (eccentric or concentric) and at the programmed velocity (50°/s or 250°/s). All contractions were isokinetic and set to 30° range of motion. Participants were verbally encouraged to maximally activate their knee extensors during the entire range of motion during the contractions, even if the contraction characteristics were unpredictable for them. The following instructions were used: “Extend your knee as forcefully as you can. You will experience that the lever will randomly move either into the direction of knee extension or flexion, and either with low or high velocity, which you will not know in advance. When it moves into knee extension, extend your knee with full effort. When it moves to knee flexion, try to resist it with full effort.”

In the KML condition, we told subjects the type of muscle contraction they will experience. We used this condition as control relative to the condition when subjects did not know the type of oncoming contraction. Our aim in the KML condition was to reduce the number of loading variations as much as possible. Because eccentric and concentric contraction are unique in nature, we kept these contractions, but we reduced velocity variations. The contraction characteristics in the KML condition were as follows: eccentric at 150°/s (EC150), and concentric at 150°/s (CC150) angular velocity. The 150°/s was used as an average of 50 and 250, used in the UML condition. In contrast with UML, participants in the KML condition received information about the contraction characteristics before each contraction. The order of contractions in the KML were as follows: 8 repetitions of CC150 and EC150, respectively, giving a total of 16 repetitions in 2 sets. The instructions on effort were the same as described in the UML condition.

Before the actual practice session, participants in both practice conditions performed one submaximal effort trial of each contraction variation at approximately 80–90% of their perceived maximal voluntary effort, allowing them to experience the possible directions and velocities of the dynamometer’s lever before the actual practice protocol. Using the joint angle, time, and torque data recorded by the dynamometer, mechanical work (expressed in J) was immediately calculated and displayed by the dynamometer’s software for each contraction [27]. 

### Data processing and statistical analyses

**Fig. 2:**
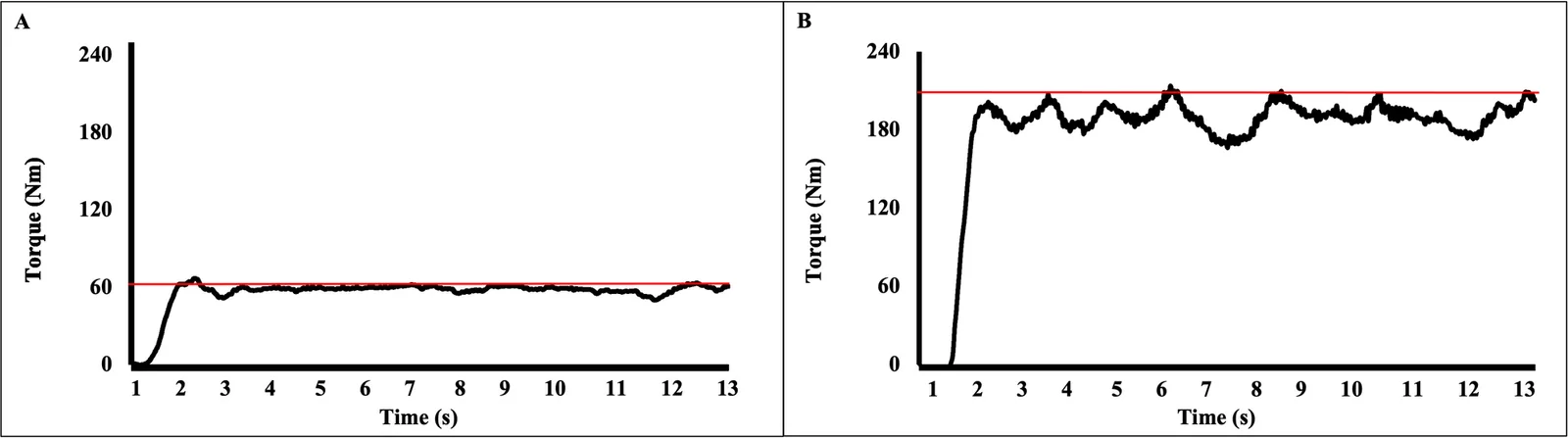
Representative torque-time recordings during TS tests performed at 20% (**A**) and 70% (**B**) of MVIC torque during knee extension with visual feedback

A representative torque-time curve recorded during the TS test is shown in Fig. 2. We calculated the absolute and the variable errors for both JPS and TS [28]. Torque values with respect to time were sampled at 1000 Hz frequency. The recorded data underwent conversion to Excel format, and all analyses were performed offline. Absolute error indicates the difference between the target and the attained values.

$$\mathrm{Absolute}\;\mathrm{error}\;=\;\left|\mathrm{Xparticipant}\;-\;\mathrm X\;\mathrm{target}\;\mathrm{value}\right|$$

The variable error was calculated as the overall standard deviation of the difference between each participant’s value and the target value.

$$\mathrm{Variable}\;\mathrm{error}\;=\;\sqrt{\sum\left(X\;participant\;-\left(\;X\;target\;value\right)\right)^2}$$

All data were checked for normality (Shapiro-Wilk test). Variables that failed normality test were log-transformed and re-checked for normality. The analyses were then done on the transformed data but we report in tables and figures non-transformed, original values.

To test the interaction between condition (UML, KML) and time (pre and post), a two-way repeated-measures ANOVA was performed for MVIC torque, JPS, and TS errors. Significant interactions were further explored using Šidák post-hoc tests.

For the analysis of trial-to-trial changes in mechanical work within each contraction variation, one-way repeated-measures ANOVA or Friedman test was used, depending on the distribution of the data. The same procedure was followed for the comparison of the average mechanical work (i.e. the mean obtained from all trials) among contraction variations. When significant effects were detected, Tukey (for ANOVA) or Dunn post-hoc tests (for the Friedman test) were applied.

A paired-samples t-test was used to compare the averaged mechanical work between the UML and KML practice conditions. Statistical analyses were performed using GraphPad Prism 9.5.1 software, and statistical significance was set at *p* < 0.05 for all analyses.

## Results

### JPS, MVIC torque, TS

Table 1 presents the acute effects of practicing with a set of unknown vs. known muscle loadings on joint position sense, knee extension MVIC torque, and torque steadiness.

**Table 1:** Joint position sense (JPS), knee extensor maximal voluntary isometric contraction (MVIC) torque, and torque steadiness (TS) before (Pre) and after (Post) a set of contractions with unknown (UML) vs. known (KML) muscle loadings

	Condition	Pre	Post
JPS: Absolute error (**°**)	UML	3.97 ± 2.13	3.95 ± 1.56
	KML	3.87 ± 1.77	3.56 ± 1.55
JPS: Variable error (**°**)	UML	3.91 ± 2.10	3.36 ± 0.79
	KML	3.59 ± 2.11	3.15 ± 0.99
MVIC torque (Nm)	UML	271.02 ± 45.29	274.15 ± 45.02
	KML	273.19 ± 43.49	276.24 ± 54.26
TS 20%: absolute error (Nm)	UML	1.88 ± 1.01	1.66 ± 0.70
	KML	2.01 ± 1.04	1.93 ± 0.95
TS 20%: variable error (Nm)	UML	1.92 ± 0.80	1.64 ± 0.54
	KML	2.07 ± 0.85	1.99 ± 0.70
TS 70%: absolute error (Nm)	UML	7.65 ± 2.52	8.06 ± 3.04
	KML	10.43 ± 4.93	8.47 ± 4.10
TS 70%: variable error (Nm)	UML	7.49 ± 1.70	7.89 ± 2.77
	KML	9.38 ± 3.09	8.09 ± 2.73

Values are mean ± standard deviation

Table 2 shows the ANOVA results. The ANOVA tests revealed no main effects for time or condition or time by condition interactions (all *p* > 0.05) for absolute or variable error JPS, absolute or variable error in TS20% and absolute error in TS70%, and MVIC torque (all *p* > 0.05). For TS70% variable error, there was a time-by-condition interaction (F_1,36_=4.729, *p* = 0.036, partial η^2^ = 0,116). Post-hoc test revealed a significant, 13.8% pre-to-post reduction in variable error in TS70% in the KML (*p* = 0.049).

**Table 2:** Condition by time interactions, main effects, and effect sizes for testing the acute effects practicing with unknown (UML) vs. known (KML) muscle loadings on joint position sense (JPS), knee extensor maximal voluntary isometric contraction (MVIC) torque, and torque steadiness (TS)

	Condition	Cohen’s d	Time main effect			Interaction		
			F	p	*η* ^2^	F	p	*η* ^2^
JPS: Absolute error	UML	0.010	0.1	0.774	0.002	0.9	0.358	0.024
	KML	0.150						
JPS: Variable error	UML	0.255	0.3	0.591	0.008	0.0	0.944	0.000
	KML	0.184						
MVIC torque	UML	0.139	0.8	0.379	0.021	0.0	0.994	0.000
	KML	0.150						
TS 20%: absolute error	UML	0.241	1.5	0.232	0.039	1.1	0.304	0.029
	KML	0.138						
TS 20%: variable error	UML	0.322	1.3	0.258	0.035	0.3	0.565	0.009
	KML	0.112						
TS 70% absolute error	UML	0.137	0.9	0.355	0.023	3.5	0.067	0.089
	KML	0.483						
TS 70%: variable error	UML	0.210	1.3	0.262	0.034	4.7	**0.036**	0.116
	KML	0.460						

Effect size cutoffs: Small: d < 0.2; Medium: 0.21–0.5; Large: 0.51-1.0

### Mechanical work differences among contraction variations

**Fig. 3:**
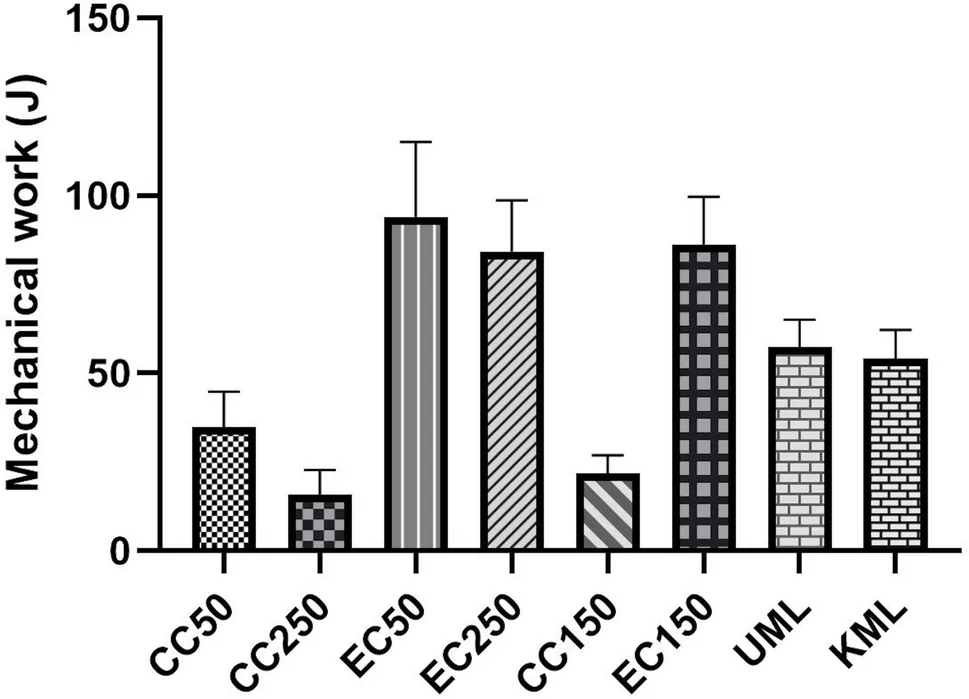
Mechanical work performed within contraction variations (all trials included), and within practice conditions (all contraction variations and trials included) during the practices (*n* = 19). Data are presented as mean ± SD

Figure 3 shows the average mechanical work performed within each contraction variation (including all trials) during the practices (both UML and KML). Because one of the six contraction variation datasets was not normally distributed, we used both parametric and nonparametric analyses. Both the one-way repeated measures ANOVA (F_4,72_=170.2, p **<** 0.001, partial η2 = 0.904) and the Friedman test (χ²(5) = 81.20, p **<** 0.001, Kendall’s W = 0.855) revealed a significant trial effect. Table 3 shows the pairwise comparisons. To express the load in UML and KML practice conditions, the mechanical work values obtained for the contraction variations were averaged within practice conditions, and we found significant difference (*p* = 0.018) between UML and KML (Fig. 3).

**Table 3:** The pairwise comparisons with corresponding p-values for mechanical works among the CC50, CC250, EC50, EC250, CC150, and EC150 contraction variations

Contraction variations	CC250	EC50	EC250	CC150	EC150
CC50	**0.027**	**< 0.001**	**< 0.001**	**0.005**	**< 0.001**
CC250		**< 0.001**	**< 0.001**	> 0.999	**< 0.001**
EC50			0.063	**< 0.001**	0.207
EC250				**< 0.001**	0.983
CC150					**< 0.001**

### Trial to trial adaptation in mechanical work

For testing the trial-to-trial adaptation during the practice, we used the mechanical work values obtained from the trials within a contraction variation. Specifically, values within each contraction variation (CC50, CC250, EC50, and EC250 in UML; CC150, and EC150 in KML) occurring at first (T1), second (T2), third (T3), and fourth (T4) in the given set, were considered for testing the trial effect (Table 4). Note that though in KML, there were eight repetitions per contraction variation, there were only four repetitions per contraction variation in UML. That is why only the first four repetitions within each contraction variation were considered for the analysis. Accordingly, we found significant trial effect for CC50 (χ²(3) = = 17.46, p **<** 0.001) in the UML, and for CC150 (F_3,54_=17.73, p **<** 0.001) and EC150 (χ²(3) = 15.32, p **=** 0.002) in the KML condition. The post-hoc tests revealed that from T1 to T4, subjects improved mechanical work in CC50 (p **<** 0.001), CC150 (p **<** 0.001), and EC150 (*p* = 0.007) contraction variations.

**Table 4:** Knee extensor mechanical work measured during the first, second, third, and fourth trial (T1 to T4) per contraction variation within unknown muscle loading (UML) and known muscle loading (KML) conditions

	T1	T2	T3	T4	trial effect	
					F/χ²	*p*
UML						
CC50	29.14 ± 13.21	32.52 ± 8.59	35.82 ± 14,02	42.20 ± 13.06	χ²=17.5	**< 0.001**
CC250	14.13 ± 2.24	14.31 ± 1.89	17.24 ± 13.71	17.88 ± 14.83	χ²=2.4	0.488
EC50	92.30 ± 17.05	94.43 ± 18.29	93.95 ± 26.99	95.78 ± 27.61	χ²=6.4	0.093
EC250	86.51 ± 15.07	85.37 ± 15.08	80.96 ± 17.93	84.57 ± 16.34	χ²=5.5	0.141
KML						
CC150	15.22 ± 3.52	19.36 ± 6.14	21.29 ± 5.96	22.65 ± 6.02	F = 17.7	**< 0.001**
EC150	77.15 ± 19.18	80.27 ± 14.08	86.72 ± 15.78	84.95 ± 13.38	χ²=15.3	**0.002**

Values are mean ± standard deviation

*CC*  Concentric contraction, *EC*  Eccentric contraction, numeric values next to each contraction abbreviation show the corresponding constant angular velocities (°/s). Note: In the case of the Friedman ANOVA (i.e. for CC50), mean and standard deviation values were used for the sake of comparability among contraction types

## Discussion

Using a novel methodological approach in the present study, we examined the immediate effects of practicing with eccentric and concentric quadriceps contractions performed at different speeds and organized into unknown (UML) and known (KML) sequences on knee extension torque, torque steadiness, and JPS in healthy young adults. While torque steadiness at 70% of MVIC improved after KML, KML and UML practice produced no other acute adaptations. We observed a contraction type- and condition-specific trial-to-trial adaptation in mechanical work after KML and UML practice.

We observed no acute adaptation to KML or UML in joint position sense. The absence of time effects and interactions in both absolute and variable JPS errors may be related to the passive nature of the joint repositioning task. Because the dynamometer moved the limb, the task relied predominantly on afferent proprioceptive input without active muscle contraction. Previous studies have shown that muscle spindle sensitivity and Golgi tendon organ contribution differ between active and passive movements, and that the lack of efference copy and decreased γ-motoneuron drive during passive motion may limit the sensitivity of joint position sense to detect adaptations over time [16, 31, 32]. Consequently, the task was not sufficiently sensitive to detect adaptations occurring in proprioceptive processing, and perhaps the use of active joint position sense test would have been more appropriate.

MVIC represents a muscle’s ability to generate maximal force in a static condition. The absence of changes in MVIC torque after KML or UML practice indicates that the practice conditions did not produce fatigue or an additional warm-up effect, beyond the general warm-up performed before MVIC testing. Practice volume and recovery period between sets were intentionally adjusted to avoid fatigue in the present study [33]. Post-activation potentiation (PAP) is considered a typical source of acute increases in MVIC force, particularly following high-intensity contractions. Although no increase in MVIC force was observed in the present study, and no EMG measurements were conducted, we found some contraction-specific trial-to-trial adaptations in both practice conditions. Specifically, mechanical work increased from trial 1 to trial 4 at CC50 contraction variation in the UML condition, and at CC150 and EC150 contraction variations in the KML condition. This, on one hand, indicates that from trial to trial, individuals adapted quickly to concentric demands, and this adaptation was also present when subjects were unaware of the nature of the upcoming contraction. However, no trial-to-trial adaptation was evident in the CC250, the EC50, and the EC250 contraction variations in the UML condition, suggesting that subjects were unable to either respond rapidly to high-velocity concentric demands or generate sufficient force during active muscle stretch, the latter likely being constrained by neural inhibition as a protective mechanism [34]. The presence of trial-to-trial adaptation only in selected contraction conditions suggests that motor adaptation is not driven solely by uncertainty, but rather by an optimal interaction between contraction type, angular velocity, and sensorimotor processing demands. The trial-to-trial increases in mechanical work observed in the KML practice condition likely reflects performance optimization driven by reduced uncertainty, rather than error-based motor adaptation. When contraction type was known in advance, participants were able to rely on predictive feedforward control and internal models, allowing progressive increases in energy output at an optimal angular velocity [35]. The only increase observed in the CC50 contraction variation within the UML condition likely reflects rapid, non-specific neuromuscular adaptation rather than task-specific learning due to the unpredictable sequence. Sensory feedback is noisy and delayed, which may impair movement accuracy and stability, however slower contraction velocity provides a longer time window for feedback processing and error correction, thereby improving motor output across trials [36]. In contrast, faster contractions may limit these feedback-driven adjustments due to increased sensorimotor noise and decreased processing time. Evidence for the effects of contraction history and velocity on post-activation potentiation also exists [37, 38], which supports our findings showing a lack of mechanical adaptation in isometric compared with certain dynamic contractions. Finally, the specific range of motion (20–50 degrees) used in the present concentric contractions falls outside the optimal knee angle (50–80 degrees), thereby providing an unusual mechanical stimulus and possibly promoting trial to trial adaptations in concentric torque [39].

Torque steadiness improved only after practicing with KMLs, and only in the 70% variable error. In torque steadiness tests, absolute and variable error represent two distinct outcomes that may reflect different regulatory control mechanisms. Absolute error reflects the participant’s ability to accurately reproduce force, whereas variable error determines the steadiness of the reproduced forces [40]. Glass and Wisneski evoked balance perturbations, and found that six practice sessions of squats on water-filled tubes improved force steadiness measured by EMG in the instability training group [41]. Others demonstrated that a single session of perturbation-based balance training acutely improved gait stability in healthy older adults [42], and a single session of 32 unpredictable treadmill-induced slips increased reactive balance control by improving the magnitude of post-perturbation responses [43]. Despite that these studies did not measure force steadiness, the results imply the rapid improvements in force control. In our study, which excluded any visual and vestibular cues during the practices, we found that torque steadiness improvement was confined to the KML practice condition. This suggests that practicing with predictable contractions may induce an immediate effect on torque steadiness, probably because the neuromuscular system can tune activation patterns more effectively, i.e., adjusts optimal motor unit recruitment and synchronization, necessary for fine motor control. The selective improvement of variable versus absolute error implies that practicing with KMLs decrease force fluctuations rather than retrains the absolute calibration of force output. Other studies examining chronic effects have reported reductions in both absolute and variable errors [40], whereas in another study, a reduction in absolute error was observed while variable error remained unchanged [44].

The improvement in variable error observed at 70% of MVIC, but not at 20%, may be explained by differences in task sensitivity and motor unit recruitment. The 20% condition likely represents a low-sensitivity task, whereas 70% MVIC requires the recruitment of larger motor units. Given that the contractions performed during the UML and KML practice protocols required maximal effort, the recruitment of larger motor units may have been selectively optimized, leading to improvements specifically at higher force levels [45].

Because of the uniqueness of the present practice protocol, i.e., the applied eccentric and concentric contraction modes in a mixed manner, the findings should be interpreted with caution due to several limitations. The most important limitation is that in the KML condition, we reduced the number of loading variations as much as possible to increase sensitivity for detecting differences between KML and UML practice conditions. Also, we detected a statistically significant, although, from practical aspect, negligible mean difference (3.2 J) in total mechanical work between KML and UML. Furthermore, we did not conduct EMG measurements to justify neural changes, especially contraction type-specific increases of post activation potentiation. Finally, instructions during UMLs and KMLs such as “extend/resist as forcefully and rapidly as possible” rather than simply “as forcefully as possibly” would perhaps have encouraged participants to activate their knee extensors more when fast practice contractions were delivered. Future studies should also examine the rate of torque development, as it may adapt to similar perturbations.

In conclusion, the acute effects of using KMLs and UMLs are limited, as neither condition improved maximal voluntary isometric torque or joint position sense, and only KML improved torque steadiness, suggesting more efficient motor unit control. However, the trial-to-trial adaptations in mechanical work mostly during KML suggest rapid neuromuscular adjustments influenced by load predictability, rather than uncertainty. Based on these findings, practitioners should prioritize high-intensity predictable loadings to immediately sharpen dynamic contractility, and motor unit control as the neuromuscular system responds more effectively to known rather than unknown mechanical demands. However, future long-term training and rehabilitation protocols should explore whether systematically transitioning from predictable to unpredictable loadings can bridge these acute gains to better prepare athletes and patients for realistic movement perturbations.

## Data Availability

The datasets generated and analyzed during the current study are available in the Zenodo repository [https://doi.org/10.5281/zenodo.20080858].

## Abbreviations

UML: Unknown muscle loading
KML: Known muscle loading
JPS: Joint position sense
MVIC: Maximal voluntary isometric contraction
TS: Torque steadiness
EC50: Eccentric contraction at 50°/s angular velocity
EC250: Eccentric contraction at 250°/s angular velocity
CC50: Concentric contraction at 50°/s angular velocity
CC250: Concentric contraction at 250°/s angular velocity
EC150: Eccentric contraction at 150°/s angular velocity
CC150: Concentric contraction at 150°/s angular velocity
EMG: Electromyography
ANOVA: Analysis of variance
